# Electrochemical Detection of Serum Antibodies Against *Mycobacterium avium* Subspecies *paratuberculosis*

**DOI:** 10.3389/fvets.2021.642833

**Published:** 2021-03-09

**Authors:** Kaoru Hatate, J. Hunter Rice, Karsten Parker, J. Jayne Wu, Amy Turner, Judith R. Stabel, Shigetoshi Eda

**Affiliations:** ^1^Department of Forestry, Wildlife and Fisheries, University of Tennessee Institute of Agriculture, Knoxville, Knoxville, TN, United States; ^2^Department of Microbiology, University of Tennessee, Knoxville, Knoxville, TN, United States; ^3^Department of Electrical Engineering and Computer Science, University of Tennessee, Knoxville, Knoxville, TN, United States; ^4^Agricultural Research Service, United States Department of Agriculture, National Animal Disease Center, Ames, IA, United States

**Keywords:** Johne's disease, diagnosis, antibody, immunoassay, electrochemical sensing

## Abstract

*Mycobacterium avium* subsp. *paratuberculosis* (MAP) causes a chronic inflammatory intestinal disease, called Johne's disease (JD) in many ruminants. In the dairy industry, JD is responsible for significant economic losses due to decreased milk production and premature culling of infected animals. Test-and-cull strategy in conjunction with risk management is currently recommended for JD control in dairy herds. However, current diagnostic tests are labor-intensive, time-consuming, and/or too difficult to operate on site. In this study, we developed a new method for the detection of anti-*M. paratuberculosis* antibodies from sera of *M. paratuberculosis-*infected animals. *M. paratuberculosis* antigen-coated magnetic beads were sequentially reacted with bovine serum followed by a horseradish peroxidase (HRP)-labeled secondary antibody. The reaction of HRP with its substrate was then quantitatively measured electrochemically using a redox-active probe, ferrocyanide. After optimization of electrochemical conditions and concentration of the redox-active probe, we showed that the new electrochemical detection method could distinguish samples of *M. paratuberculosis-*infected cattle from those of uninfected cattle with greater separation between the two groups of samples when compared with a conventional colorimetric testing method. Since electrochemical detection can be conducted with an inexpensive, battery-operated portable device, this new method may form a basis for the development of an on-site diagnostic system for JD.

## Introduction

A bacterial pathogen, *Mycobacterium avium* subsp. *paratuberculosis* (MAP) infects the intestinal tissue of animals and causes a chronic wasting disease called Johne's disease (JD). JD affects domestic animals such as farm deer, sheep, and dairy cattle. The prevalence of JD in cattle in Australia, New Zealand, Europe, and the United States was estimated to range from 10 to 60% (1–4). United States Department of Agriculture (USDA) reported that 68.1% of dairy herds in the US are contaminated with MAP, and the most recent report estimated that the actual herd-level prevalence of MAP was higher than 90% (5). Clinical symptoms of JD include submandibular edema, emaciation, severe diarrhea, and reduction in milk production (6). Economic loss caused by JD in dairy herds is mostly due to the reduced milk production and premature culling of JD-affected cattle (7). In the USA, JD causes an estimated annual loss of $220 million to the agricultural economy (4, 8–10).

The current recommendation for JD control in dairy herds involves test-and-cull strategies in conjunction with risk management and intervention (11, 12). However, multiple mathematical modeling studies have suggested that this approach (test-and-cull) would require long-term effort, investment, and widespread compliance to effectively reduce JD prevalence in dairy herds (13–15). In a 6-years field study, the prevalence of JD in 9 dairy herds was reduced from 11.6 to 5.6% by applying ELISA-based control measures, but no statistically significant difference could be detected across the same population when MAP prevalence was estimated by the slower but more sensitive fecal culture test (16). Therefore, the test-and-cull approach itself is unlikely a cost-effective control measure and should be accompanied by the prevention of within-farm MAP transmission.

The fecal-oral mode of transmission has been identified as the most important route of infection for JD, while the contribution of alternative routes, such as infection through MAP-contaminated milk or colostrum, has more slowly gained attention (17, 18). Young animals (1-year-old or younger) are known to be especially susceptible to MAP infection (19–21), and a longitudinal study demonstrated JD in dairy farms could be reduced by preventing infection of young animals (calves and heifers) through feces and milk/colostrum (16). To achieve this, the results of ELISAs were used to inform animal segregation based on disease state and for the selection of MAP-free colostrum for feeding. A recent study showed a direct correlation between ELISA-positivity and rate of shedding of MAP into milk (up to 10^6^/ml of milk) (22), supporting the strategy of using ELISA results for the prevention of MAP transmission to young animals.

Currently, ELISA tests for JD are conducted in diagnostic laboratories, incurring long turnaround time (days to a week) and excess labor/shipment costs. Thus, the effectiveness of the aforementioned ELISA-based JD risk management measures could significantly be improved if all the steps of the assay were performed at the site of sample collection, on the farm. Also, since JD is often mischaracterized as an infection with another type of enteric pathogen, on-site diagnosis of JD could reduce unnecessary treatment of the animal with antibiotics. This study aims to develop a platform technology for rapid, on-site detection of antibodies against MAP for JD diagnosis. The principle of antibody detection in our method is based on the conventional ELISA, but for implementation into an inexpensive portable device, we utilized a magnetic-bead-based immunoassay (23) and a new electrochemical method for antibody detection.

The last step of the ELISA test is to quantify the reaction of enzyme tagged to the secondary antibody. A widely used enzyme is horseradish peroxidase (HRP), and the quantification of HRP can be done by measuring color, fluorescence, or chemiluminescence produced by HRP-substrate reaction. Since such detection requires bulky and/or expensive equipment, electrochemical detection approaches have been evaluated in previous studies. This study demonstrated that the analytical sensitivity of the new electrochemical detection of HRP is comparable to a conventional optical method. Further, we tested the new electrochemical method for detecting anti-MAP antibodies in serum samples of JD-affected (MAP-infected) animals using magnetic beads for antibody binding future automation as described in our previous work (23).

## Materials and Methods

### Reagents

A solution of 10 mM phosphate-buffered saline (pH 7.4, PBS) supplemented with 0.05% (v/v) surfactant Tween-20 (Thermo Fisher Scientific, Waltham, MA, USA) served as the wash solution (PBST). A buffer for serum dilution and blocking (Buffer B) was prepared by adding SuperBlock blocking solution (10 v/v%, Thermo Fisher Scientific/Pierce, Waltham, MA, USA) and Tween 20 (0.05 v/v, % Thermo Fisher Scientific, Waltham, MA, USA) to 10 mM (pH 7.4, PBS). Bovine serum albumin (BSA, Sigma-Aldrich, St. Louis, MO, USA) solution (1 w/v%) was prepared in PBST. HRP-labeled goat anti-bovine immunoglobulin G (H+L) obtained from Jackson ImmunoResearch (West Grove, PA, USA) was diluted in Buffer B immediately before use. Potassium ferrocyanide {K_4_[Fe(CN)_6_]} and potassium ferricyanide {K_3_[Fe(CN)_6_]} purchased from Thermo Fisher Scientific/ACROS Organics (Waltham, MA, USA) were stored dry and prepared fresh before each experiment. Purified HRP enzyme and 3,3′, 5,5′-tetramethylbenzidine (TMB) substrate were purchased from Thermo Fisher Scientific (Waltham, MA, USA). Other chemicals such as acetone and 2-propanol were purchased from Thermo Fisher Scientific/Fisher Chemical (Waltham, MA, USA).

### Serum Samples

A total of 15 bovine serum samples were obtained from USDA-National Animal Disease Center (NADC; Ames, IA, USA). The samples had been collected from Holstein dairy cows purchased from dairy farms in the USA (IA, MN, and ND) from 2014 to 2019. Among the 15 samples, five samples were collected from female Holstein cattle (3–12 years old, mean ± SD = 5.2 ± 3.8) that had been diagnosed as negatives for JD by interferon-gamma, ELISA, and fecal culture test. All the negative samples were from animals maintained separately in the dairy barn at the USDA-NADC. The other 10 samples were collected from female Holstein cattle (4–8 years old, mean ± SD = 5.8 ± 1.3) that had been diagnosed as JD positives by interferon-gamma, ELISA, and fecal culture test. Among the 10 infected animals tested in this study, five of them were shedding over 100 MAP/g of feces, four were shedding <100 (one of them was only <3 MAP/g feces), and the other one was unknown (culture contaminated). HyClone™ fetal bovine serum (FBS, GE Healthcare, Wauwatosa, WI, USA) was also used as a negative reference serum, and a pool of JD-ELISA (IDEXX)-positive bovine samples obtained from BioVet Inc. (called BV+, Beaudry Saint-Hyacinthe, Canada) was used as a positive reference serum. All serum samples were diluted 1:1 with glycerol and stored at −20°C until use.

### Antibody Detection Assay

***a. Ethanol-Vortex ELISA Test***

Ethanol-Vortex ELISA test (EVELISA) was conducted as described in the previous report (24). Briefly, a 96-well plate was prepared by an overnight immobilization of 50 μl ethanol-extracted MAP antigen and then blocked with Buffer B for 1 h, before 50 μl of diluted serum samples (1:100 dilution in Buffer B) were inoculated for 30 min at room temperature. After washing the wells four times with PBS-T, 50 μl of HRP-labeled goat anti-bovine secondary antibody (diluted 1:1,000 in Buffer B) was added and incubated at the room temperature another 30 min. After four more washes with PBST, 100 μl of TMB substrate solution was added to each well, and optical density (OD) was measured at 655 nm after 16 min of reaction time had elapsed.

***b. Bead-Based Antibody Detection Assay***

**Figure 1:**
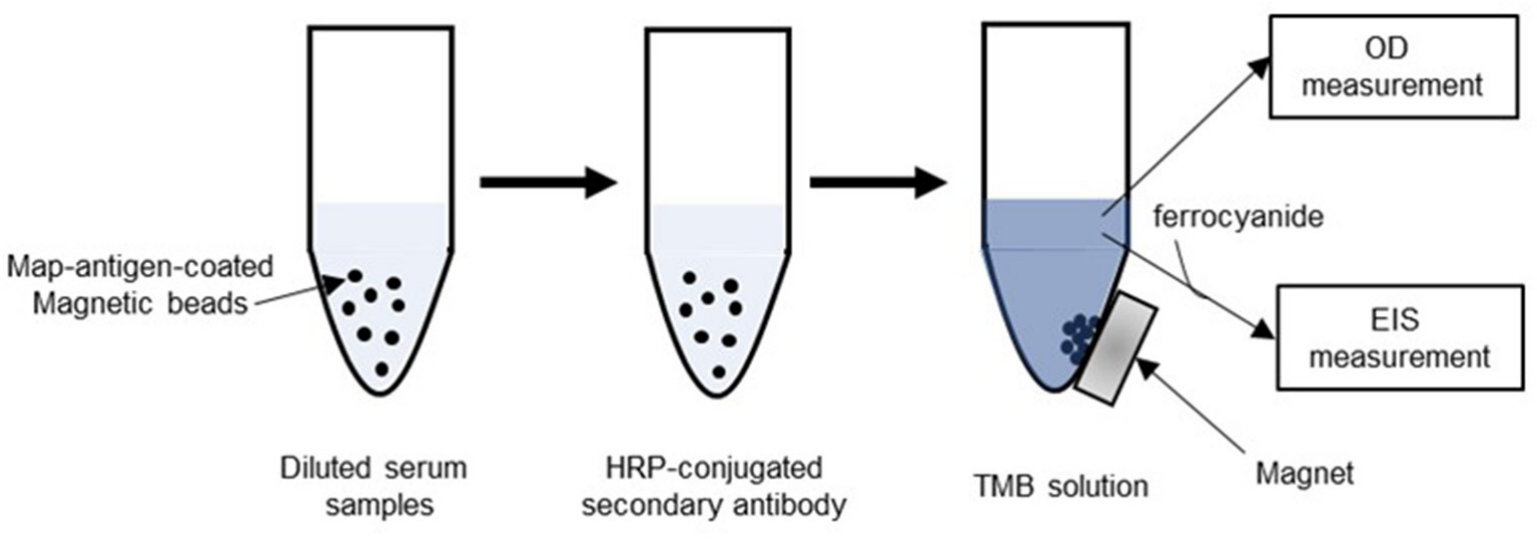
Schematic of bead-based antibody binding assay. Magnetic beads coated with antigen of *Mycobacterium avium* subsp. *paratuberculosis* (MAP) were treated with diluted bovine serum samples from Johne's disease (JD)-positive and negative animals. After removal of the serum solution, the beads were then incubated with secondary antibody (anti-bovine immunoglobulin antibody) conjugated with horseradish peroxidase (HRP). After washing, the beads were reacted with 3,3′,5,5′-tetramethylbenzidine (TMB) followed by a 6-min incubation. The TMB solution was subjected to the colorimetric or electrochemical measurement. For the electrochemical measurement, a ferrocyanide solution was added to the TMB solution prior to the measurement.

The principle of the assay used in this study is depicted in Figure 1. In brief, MAP-antigen-coated magnetic beads were incubated with a bovine serum samples from a JD-infected or uninfected animal, and bound antibody was labeled with an HRP-tagged secondary antibody. After washing, the HRP-tagged secondary antibody remaining on the beads was detected either calorimetrically (OD measurement) or electrochemically [electrochemical impedance spectroscopy (EIS) measurement]. The detailed procedures are described below (***b-1***and ***b-2***).

***b-1. Bead-Based Antibody Binding Assay***

The preparation of 80% ethanol extract (EE) of MAP Linda (human isolate) antigen was conducted as described previously (25, 26), and the protocol for antibody reactions on magnetic beads was similar to our previous work (23). Polystyrene coated magnetic beads (∅4.13 μm, SpheroTech, Lake Forest, IL, USA) were suspended in diluted MAP EE (1:40 in absolute ethanol) with gently agitated on a shaker overnight at room temperature. The antigen-coated beads were blocked by 1% BSA for 30 min and the magnetic separation rack (Thermo Fisher Scientific, Waltham, MA) was used by acclimating the beads to replace supernatant with 100 μl of diluted serum samples (1:50 in 1% BSA in PBST). After 30 min incubation at room temperature, the supernatant was removed and replaced with 100 μl of diluted HRP-labeled goat anti-bovine secondary antibody (1:500 in 1% BSA in PBST) on the magnetic rack. Again for 30 min incubation on a shaker, the beads were washed with 500 μl of PBST four times and a half portion of the last wash was left for the OD measurement, and the other half was left until electrochemical measurement.

***b-2. Measurement of HRP Activity***

**b-2-1. Optical Density Measurement**

To determine the limit of detection (LOD) for electrochemical measurement described below, the OD of the TMB solution reacted with different concentrations of HRP was compared with those measured by the electrochemical measurement. The A series of diluted HRP solutions was prepared by serially diluting HRP with PBST to make 1, 2, 4, 8, and 16 pM (the final concentrations). Fifty microliters of each HRP solution were reacted with 100 μl of TMB substrate in a 96-well plate for 6 min, and OD value was read at 655 nm using a microplate reader (Bio-Rad, Hercules, CA). Simultaneously, the electrochemical reading of the same samples was performed as described below. Each measurement was performed in triplicate. Similarly, the OD (655 nm) was measured after reacting a TMB solution with magnetic beads prepared as described in the Bead-based antibody binding assay section.

**b-2-2. Electrochemical Measurement**

A portable potentiostat (Autolab, PGSTAT204, Metrohm, Riverview, FL, USA) in coordination with NOVA software was used for the EIS measurement. Gold interdigitated microelectrodes (IDE, printed on a ceramic substrate with 100 mm gaps, Metrohm, Riverview, FL, USA) were immersed in an acetone bath and rinsed with 2-propanol and purified water (>18 MΩ × cm) sequentially before use. Before measurement, the impedance of a solution of 5 mM ferrocyanide and ferricyanide mixture was recorded to ensure the quality (cleanness) of each IDE microelectrode. IDE electrodes that gave 500 Ω or less on the imaginary axis at the peak of the hemisphere on the Nyquist plot were used for the assay. After the sample was reacted with TMB substrate for 6 min in the dark, a ferrocyanide solution was added to the sample (2–20 μl of the sample) to make the concentration indicated in each figure before loading onto the IDE microelectrode. The impedance was recorded at frequencies ranging from 100 to 10 kHz. The IDE microelectrode was rinsed thoroughly with purified water and air-dried between readings. Each measurement was performed in duplicate. For EIS analysis, the values of charge transfer impedance (R_ct_) were calculated by using the NOVA software.

The Rct of a control buffer was used to normalize Rct of the tested samples. Reciprocal of the ratio (RR) was calculated by dividing the Rct of a control buffer [a mixture of 20 μl TMB buffer and 2 μl ferrocyanide solution [50 mM]] by that of each tested sample. The reciprocal value was used since Rct decreases as the HRP–TMB reaction proceeds.

(1) $$\begin{array}{l} RR=\left\{\frac{Rct\;of\;the\;control\;buffer}{Rct\;of\;the\;sample}\right\} \end{array}$$

### Statistical Analysis

Sample-to-positive (S/P) ratios were calculated according to the following equation:

(2) $$\begin{array}{l} \text{S}/\text{P}=\left\{\frac{Value\;of\;sample\;-value\;of\;FBS\;control\;}{Value\;of\;BV^{+}control\;-value\;of\;FBS\;control}\right\}, \end{array}$$

where “value” refers to either RR or OD. Pearson's correlation analysis and Mann–Whitney *U* test were performed in Excel and R, respectively. The LOD was estimated as described previously (27, 28). Briefly, the RR or OD values were plotted against the HRP concentrations (1–4 pM and 1–16 pM, respectively). A linear regression generated in Excel was used to find the values of slope and y-intercept of the line of best fit. The SD of the data was multiplied by 3 and added to the y-intercept, which was then used to find the LOD (i.e., the corresponding concentration of HRP on the linear regression line).

## Results

### Optimization of Assay Conditions for Electrochemical Measurement of HRP Activity

**Figure 2:**
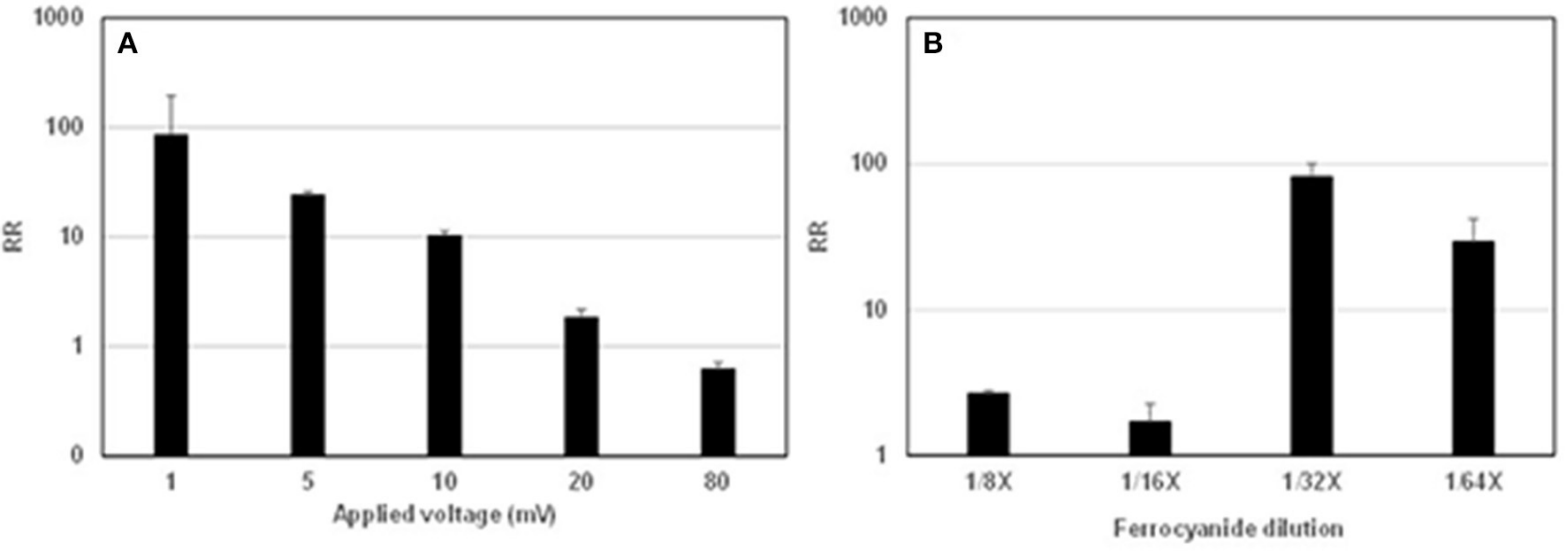
Optimization of assay conditions for electrochemical measurement of HRP activity. The y-axis represents the reciprocal ratio (RR) values calculated as described in the method section. **(A)** Different voltages were applied to the interdigitated microelectrode (IDE) at the measurement of HRP activity. The experiment was conducted with three replicates. A similar result was obtained in a separate experiment. **(B)** Concentrations of ferrocyanide [1/8X (0.6 mM), 1/16 (0.3 mM), 1/32X (0.15 mM), and 1/64 (0.08 mM)] were added to the HRP–TMB reaction solution prior to the electrochemical measurement of HRP activity.

A solution of HRP at 8 pM was used for the optimization of the electrochemical detection method. The solution was reacted with TMB solution, mixed with a ferrocyanide solution, and loaded onto an IDE microelectrode followed by an EIS measurement. In Figure 2A, different voltages were applied to the IDE microelectrode for the EIS measurement. The highest RR value was obtained at 1 mV but the variation among the triplicate measurements (SD of 109.3) was higher than that obtained at other voltages. Based on this result, 5 mV was used in the following experiments. As shown in Figure 2B, the highest RR value was obtained at 0.16 mM ferrocyanide (labeled 1/32X in the figure), and the concentration was used in the following experiments.

### Comparison of Electrochemical and Colorimetric Measurements of HRP Activity

**Figure 3:**
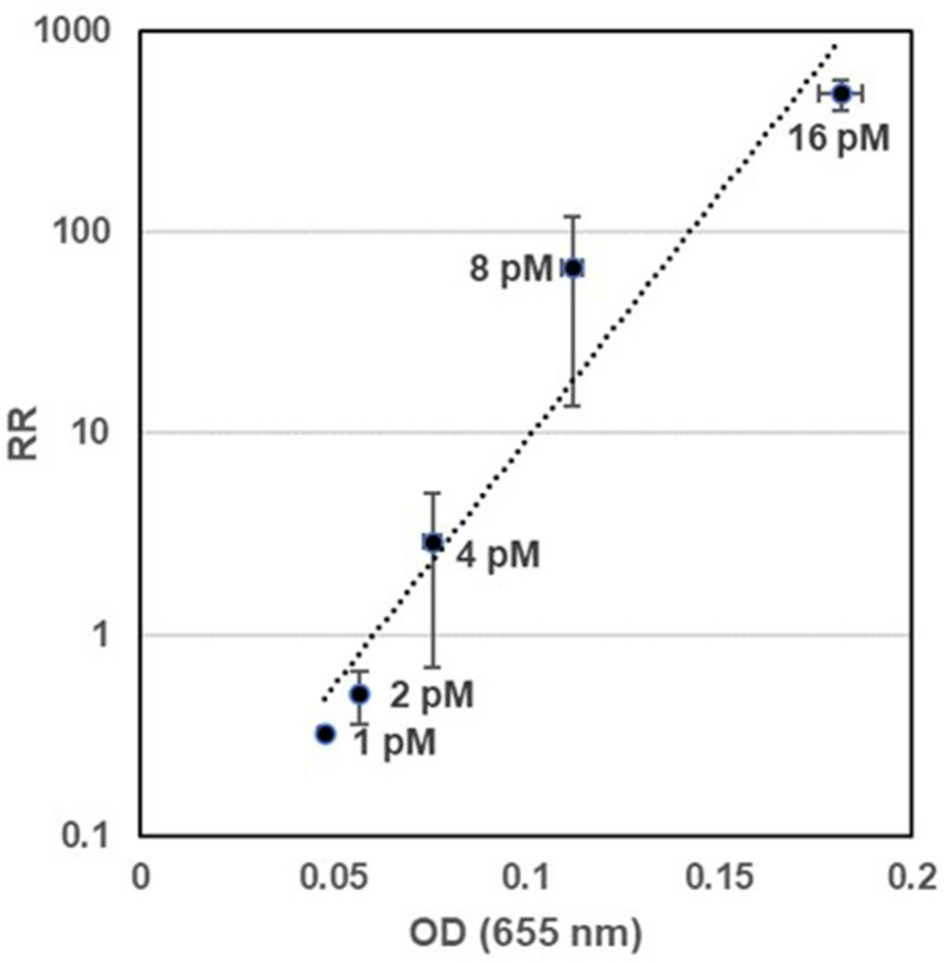
Comparison of electrochemical and colorimetric measurements of HRP activity. HRP–TMB reaction was measured using the optimized electrochemical method and a conventional colorimetric method [measurement of the optical density (OD) at 655 nm] at different HRP concentrations. HRP concentrations (pM) used in the experiment were indicated next to each dot. The experiment was conducted with three replicates. A similar result was obtained in a separate experiment.

The electrochemical measurement of HRP activity was compared with a conventional colorimetric measurement by testing different HRP concentrations ranging from 1 to 16 pM. A strong linear correlation was observed when the electrochemical (RR) and colorimetric measurements (OD) were plotted on a semi-log scattered plot (R^2^ = 0.9376, RR = 0.0331e^56.3OD^) (Figure 3). The difference of ODs at the level of 0.01 (1 and 2 pM HRP) could be differentiated by the electrochemical method with a statistical significance (*t*-test, *p* < 0.05). Under the tested condition (6 min reaction of HRP and TMB), the LODs of the colorimetric and electrochemical method for the detection of HRP were estimated to be 0.94 pM (41 pg/ml) and 1.35 pM (58.9 pg/ml), respectively.

### Comparison of Anti-MAP Antibody Detection Using the New Electrochemical Method and EVELISA

**Figure 4:**
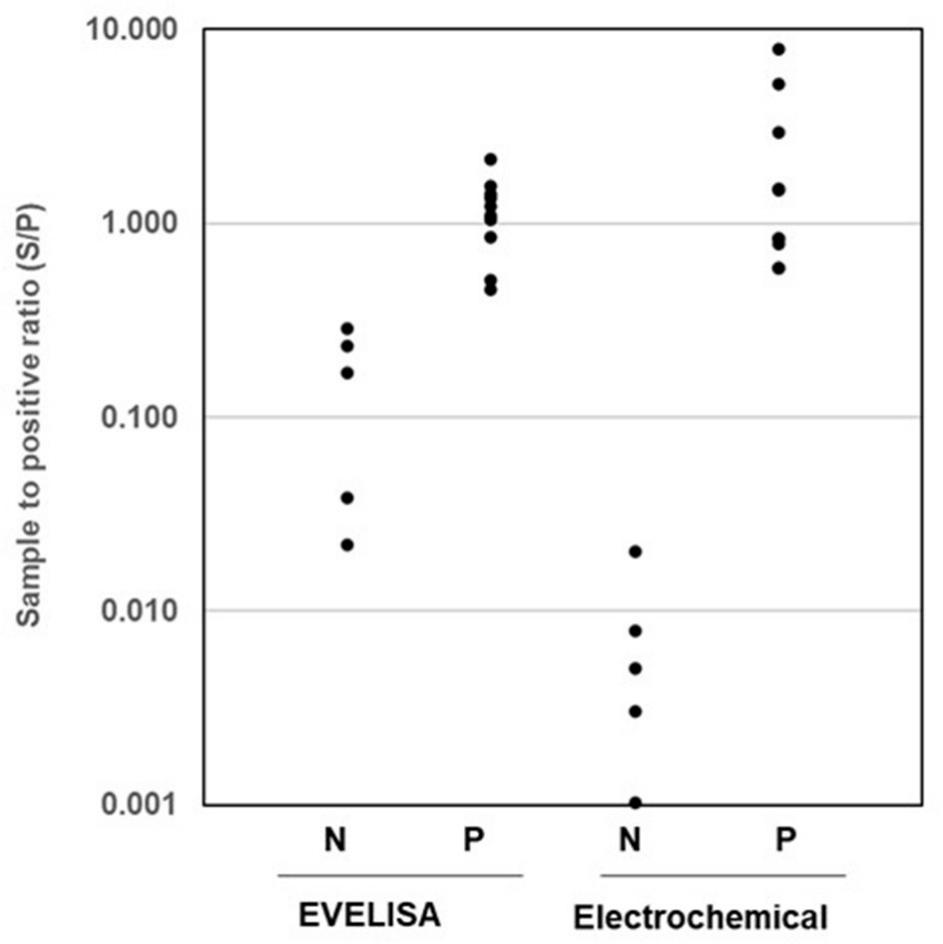
Comparison of anti-MAP antibody detection using the Ethanol-Vortex ELISA test (EVELISA) and new electrochemical method. Serum samples from 5 JD-negative (N) and 10 JD-positive (P) samples were tested by the EVLISA and the electrochemical method. The experiment was conducted with two replicates. A similar result was obtained in a separate experiment.

Fifteen bovine serum samples were collected from dairy cattle with known JD status and tested for anti-MAP antibody by using the new electrochemical method and the EVELISA test (24, 29) (Figure 4). For the electrochemical method, MAP antigen was immobilized on magnetic beads and reacted with bovine serum followed by an HRP-conjugated secondary antibody. In both methods, JD-negative (N) and JD-positive (P) samples were clearly separated, and the differences between the two groups (N and P) were statistically significant (*p* < 0.01, Mann–Whitney *U* test). As shown in the figure, the separation between the highest negative and lowest positive was greater when the samples were tested by the electrochemical method (0.02 vs. 0.586) than when they were tested by the EVELISA test (0.285 vs. 0.454).

## Discussion

Due to the lack of practical treatment for JD control, the detection and removal of JD-positive animals are recommended for the reduction of the burden of the disease on the dairy farms (30). Our group developed the ELISA test (named EVELISA) that showed a higher sensitivity for the detection of anti-MAP antibodies in serum and milk of cattle tested positive for MAP infection by the fecal culture test (29, 31, 32). However, mathematical modeling studies (15, 33, 34) indicated that ELISA-based JD control can be prohibitively costly, especially in the first few years after implementation of more stringent control measures, and the approach may not be economically feasible for many dairy farmers. Also, there is still a requirement for the ELISA procedure to be conducted in well-equipped diagnostic laboratories, causing a long turnaround time (from days to a week). Despite the drawbacks of the ELISA and the difficulties with implementing an effective MAP-management strategy, “on-site” detection of anti-MAP antibodies in serum or milk may still be useful in some scenarios, such as (1) rapid screening of animals for JD before importing them into a new dairy herd, (2) examining dams for JD status to avoid feeding calves with colostrum/milk contaminated with MAP, and (3) segregating anti-MAP antibody-positive and negative cattle within maternity pens (16). This study aimed to develop base technology for on-site detection of anti-MAP antibodies in dairy cattle by combining magnetic-bead-based immunoassay and a new electrochemical method for HRP detection.

Several studies used electrochemical methods, such as chronoamperometry (CA) and cyclic voltammetry (CV), for the detection of HRP–TMB reaction (35–38). All the studies directly detected TMB oxidized by HRP and did not use ferrocyanide. For example, Singh et al. used CA for electrochemical direct detection of oxidized TMB for the detection of serum antibodies against parasitic nematode, *Ostertagia ostertagi* (35). The antibody-antigen reaction was conducted in a regular ELISA plate and, after the reaction, the TMB-containing solution was transferred onto an electrode for the electrochemical measurement. The difference between positive and negative serum samples was found to be 1.6 times greater than an optical ELISA method. In the present study, the difference between positive and negative samples observed with the new electrochemical method (293-fold, 0.586/0.02) was much greater than that obtained with an optical ELISA method (1.6-fold, 0.454/0.285). There are several potential explanations for this improvement in our protocol including the difference in the antibody-antigen reaction format [i.e., plate-based in Singh's vs. magnetic bead-based in our study (23)]. Another difference is that our protocol included a redox reagent, ferrocyanide, to facilitate charge transfer at the surface of the electrode. Another recent study by Barhoumi et al. also utilized CA for direct detection of HRP–TMB reaction (36). In the study, a capture antibody against tumor necrosis factor (TNF)-a was immobilized onto an electrode through magnetic microparticles, reacted with a TNF-containing solution followed by reaction with a detection anti-TNF antibody labeled with HRP. After reacting with TMB, CA reading was conducted to quantify TMB oxidized by the HRP on the electrode. The LOD of the method for TNF quantification was estimated to be 0.2 pg/ml (0.01 pM). The LOD of our method for HRP detection was much higher 58.9 pg/ml (1.35 pM); however, such direct comparison may not be meaningful as (1) the secondary antibody used in the Barhoumi study may have been labeled with multiple HRP molecules, (2) TMB reaction was occurring at the electrode in their study while ours occur in a solution, and, more importantly, (3) the LOD of our method is likely improved if we extend the HRP–TMB reaction time. For direct comparison, we tested JD-negative and JD-positive samples with the new electrochemical method (impedance analysis of ferrocyanide-mediated oxidized TMB detection), which performed better than previously reported protocols (direct detection of oxidized TMB with CA or CV) and found that the new protocol differentiated the positive samples from negative samples with much greater separation than the previous protocols (Supplementary Figure 1).

By using the MAP antigen used in EVELISA, we demonstrated that a magnetic-bead-based immunoassay for anti-MAP antibody detection can be carried out in a microfluidic system (23). Magnetic-bead-based immunoassay has some advantages over ELISA, such as a high surface-to-volume ratio for antigen immobilization and compatibility with the microfluidic system. However, since our previous study utilized a fluorescent tag for the detection of secondary antibody (23), it required bulky and expensive equipment for the assay. Therefore, in this study, the new electrochemical detection method was combined with the magnetic-bead-based immunoassay we developed in the previous work. A similar concept has been used in several previous studies (36, 39, 40), but this study is unique in that the new electrochemical method was used for the diagnosis the target disease, JD. Since we tested a limited number of samples in this study, the diagnostic sensitivity and specificity cannot be discussed; however, further studies are warranted based on the observation that the separation between negative and positive samples was greater when the samples were tested by the electrochemical method than that tested by the EVELISA test. In summary, this study provides a base technology for the development of an on-site detection of serum antibodies against MAP for JD control. Once developed, the costs for the on-site detection system ($2 per sample and $200 for equipment) are expected to be less expensive than the ELISA test ($5 per sample and $5,000 for equipment).

## Data Availability Statement

The raw data supporting the conclusions of this article will be made available by the authors, without undue reservation.

## Author Contributions

SE and JR: conceptualization. KH, JR, and KP: data curation. KH, SE, JR, KP, and JW: data analysis. SE, JW, and JS: funding acquisition and supervision. JR and KH: methodology. SE: project administration. SE, JW, JS, and AT: resources. KH, SE, and JR: visualization. KH and SE: writing (original draft). KH, JR, KP, JW, AT, JS, and SE: writing (review and editing). All authors contributed to the article and approved the submitted version.

## Conflict of Interest

The authors declare that the research was conducted in the absence of any commercial or financial relationships that could be construed as a potential conflict of interest.

## Supplementary Material

Supplementary Figure 1 Comparison of **(A)** electricalchemical impedance spectroscopy (EIS), **(B)** cyclic voltammetry (CV), and **(C)** chronoamperometry (CA) for detection of oxidized TMB. JD-negative [fetal bovine serum (FBS), gray dots] and JD-positive control (BV+, black dots) samples were tested by using the new electrochemical method **(A)** with ferrocyanide and previously reported direct TMB detection with CV **(B)** and CA **(C)**. It clearly shows that the new method performed better than the previous methods in separating the positive sample from negative sample.
