# Supplementary figures and images for: Electrochemical Detection of Serum Antibodies Against Mycobacterium avium Subspecies paratuberculosis
Source: Front Vet Sci. 2021 Mar 9;8:642833. doi: 10.3389/fvets.2021.642833 (PMC8006348; doi:10.3389/fvets.2021.642833)

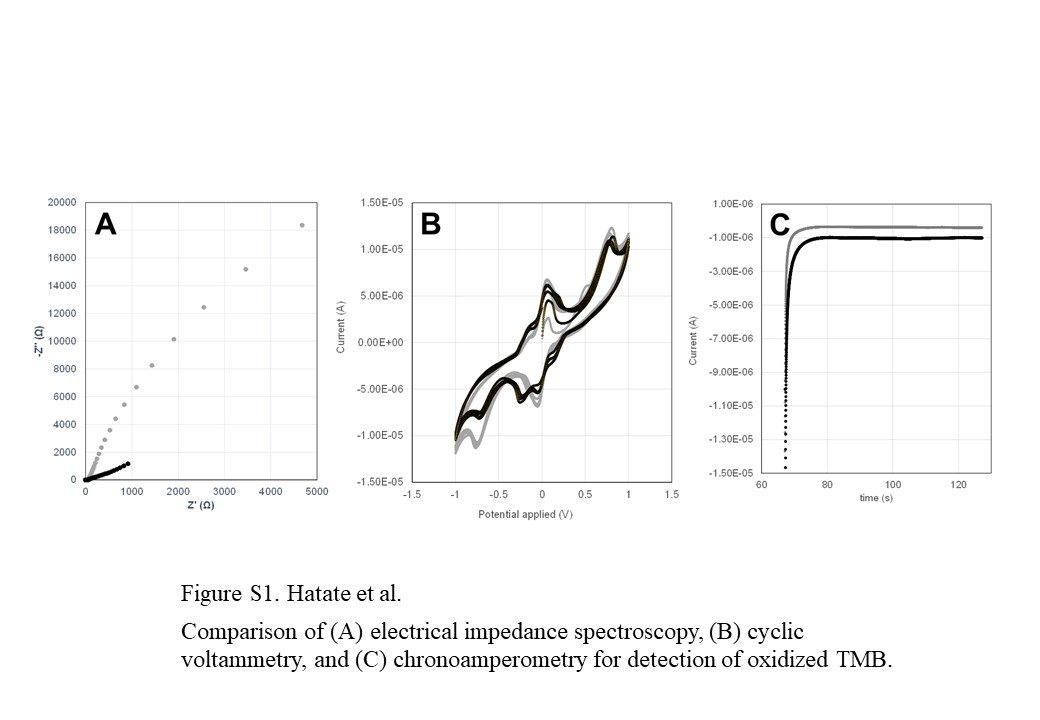

Supplement: Supplementary Figure 1 — Comparison of (A) electricalchemical impedance spectroscopy (EIS), (B) cyclic voltammetry (CV), and (C) chronoamperometry (CA) for detection of oxidized TMB. JD-negative [fetal bovine serum (FBS), gray dots] and JD-positive control (BV+, black dots) samples were tested by using the new electrochemical method (A) with ferrocyanide and previously reported direct TMB detection with CV (B) and CA (C). It clearly shows that the new method performed better than the previous methods in separating the positive sample from negative sample. [file Image_1.JPEG]
